# Assessing the Aflatoxins Mitigation Efficacy of Blueberry Pomace Biosorbent in Buffer, Gastrointestinal Fluids and Model Wine

**DOI:** 10.3390/toxins12070466

**Published:** 2020-07-21

**Authors:** Usman Rasheed, Qurat Ul Ain, Muhammad Yaseen, Sayantan Santra, Xiaohua Yao, Bin Liu

**Affiliations:** 1 Institute of Applied Microbiology, College of Agriculture, Guangxi University, Nanning 530005, China; usman4pp@gmail.com (U.R.); sayantan.santra@outlook.com (S.S.); xhyao@gxu.edu.cn (X.Y.); 2 Guangxi Key Laboratory of Petrochemical Resource Processing and Process Intensification Technology, School of Chemistry and Chemical Engineering, Guangxi University, Nanning 530004, China; qurat390@gmail.com; 3 Institute of Chemical Sciences, University of Peshawar, Peshawar, KP 25120, Pakistan; myyousafzai@gmail.com

**Keywords:** Aflatoxin, blueberry biosorbent, model wine, gastrointestinal fluid, adsorption isotherm, textural characterization

## Abstract

Blueberry (BB) and cherry pomace were investigated as new biosorbents for aflatoxins (AFs) sequestration from buffered solutions, gastrointestinal fluids and model wine. Among the tested biosorbents, BB exhibited the maximum adsorption performance for AFs and hence was further selected for the optimization of experimental parameters like pH, dosage, time and initial concentration of AFs. Material characterizations via scanning electron microscopy (SEM), Fourier transform infrared (FTIR) spectroscopy, N_2_ adsorption-desorption isothermal studies, thermogravimetric analysis (TGA) and X-ray photon spectroscopy (XPS) techniques revealed useful information about the texture and chemical composition of the biosorbents. The fitting of isothermal data with different models showed the model suitability trend as: Sips model > Langmuir model > Freundlich model, where the theoretical maximum adsorption capacity calculated from the Sips model was 4.6, 2.9, 2.7 and 2.4 mg/g for AFB1, AFB2, AFG1 and AFG2, respectively. Kinetics study revealed the fast AFs uptake by BB (50–90 min) while thermodynamics studies suggested the exothermic nature of the AFs adsorption from both, single as well as multi-toxin buffer systems, gastrointestinal fluids and model wine. Accrediting to the fast and efficient adsorption performance, green and facile fabrication approach and cost-effectiveness, the newly designed BB pomace can be counted as a promising contender for the sequestration of AFs and other organic pollutants.

## 1. Introduction

Aflatoxins (AFs), on a worldwide scale, are the most toxic class of mycotoxins primarily produced by *Aspergillus parasiticus*, *Aspergillus nomius* and *Aspergillus flavus* [1,2]. More than 200 kinds of AFs are known to occur in nature among them, most concerned toxins are aflatoxin B1 (AFB1), aflatoxin B2 (AFB2), aflatoxin G1 (AFG1) and aflatoxin G2 (AFG2) [3] which is attributed to their frequent contamination in cereals, wine, human and animal feedstuffs, water bodies and even in drinking water [4,5,6]. AFs are classified as Group 1 carcinogens by the International Agency for research on Cancer [7] with the order of toxicity as: AFB1 > AFG1 > AFB2 > AFG2 [8]. Frequently reported malformations owing to AFs exposure include hemorrhage, hepatitis, neural tube defects, stunted growth, edema, hepatic carcinoma and esophageal cancer [9]. 

AFs can survive heat and physicochemical treatments in the food processing industry, hence their removal from the food is indispensable and to attain that goal, certain conventional strategies have been demonstrated such as biodegradation, irradiation and physical separation (filtration, adsorption/biosorption and extraction etc.), among which adsorption is considered as the most economical and feasible approach [10]. Classes of materials commonly employed as mycotoxin adsorbents include inorganic clay minerals (zeolites, bentonites etc.) as well as organic materials such as yeast cell wall, activated carbon, polymeric organic compounds and processed plant fibers [11]. In the circle of adsorption research, biosorption possesses dual benefits i.e., suitable management of the bio-wastes and a great degree of greenness in the approach for the efficient removal of the pollutants [12]. Recently, agro-industrial wastes have been widely employed for the sequestration of organic and inorganic toxic substances [13,14]. Agricultural wastes are appealing bio-sorbents as their major constituents are cellulose, hemicellulose and lignin which carry abundant functional groups like hydroxyl, carbonyl, carboxylic/sulfhydryl carboxylic, phenolic, esters and amino etc. which could be superlative active sites for capture organic contaminants [15,16]. The chemistry during the biosorption includes hydrogen bonding, π–π interactions, secondary bond forces and physicochemical interactions between the functional groups i.e., carboxyl, phosphonates, phosphates, sulfhydryl, amines and amides etc. on bio-sorbents and pollutants [17]. Therefore, the presence of these functional groups renders the bio-agricultural wastes as promising materials for the adsorptive removal of organic/toxic compounds. These bio-sorbents used for the removal of pollutants include tomato processing waste [18], forest biowaste [19], cherry waste [20], apricot stone [21], and grape pomace waste [22], while specifically for mycotoxins, grape pomace waste [23], Pyracanth leaves, Aloe powder [12], *Pyracantha koidzumii* biomasses [24], banana peel [24] and grape processing waste [25] are noteworthy. Blueberry and cherry wastes (pomace) obtained from wine and juice industries could also be envisaged as efficient AFs adsorbents owing to their higher lignin, cellulose, hemicellulose contents. Cherry and berry pomace accounts for over 90% of the fruit dry matter with substantial amounts of antioxidant compounds including anthocyanins, polyphenols, flavonoids etc. Pomace is often used as animal feed, fuel, extract source with further transformation but mostly seen as waste. Therefore, using these waste materials as biosorbents would provide two-way benefits, i.e., production of high capacity AFs adsorbents owing to their cellulose- and lignin-rich nature, while the presence of higher amounts of antioxidants would help in improving the overall health of poultry.

On the other side, most of the AFs removal studies reported previously focused on AFB1 adsorption only [26], ignoring the other concurrently occurring counterparts like AFB2, AFG1 and AFG2 [2,27,28]. Keeping in mind these facts, the present study is designed to employ blueberry (BB) and cherry (CH) industrial wastes for the removal of AFs (AFB1, AFB2, AFG1 and AFG2) from the aqueous solution through systematic batch adsorption experiments and its possible application in gastrointestinal fluid and the wine industry. The pre and post adsorption adsorbents were characterized by advanced textural characterization techniques while detailed mechanism for the adsorption process of AFs over the prepared adsorbent was explained based on the adsorption results and characterization findings. Quantification of the AFs in post adsorption samples was performed by a High-Performance Liquid Chromatography (HPLC) system equipped with Waters fluorescence detector 2475 (λex 365 nm and λem 435 nm) and autosampler was used for AFs quantification. 

## 2. Results and Discussion

### 2.1. Textural Characterization of the Bio-Sorbents

**Figure 1:**
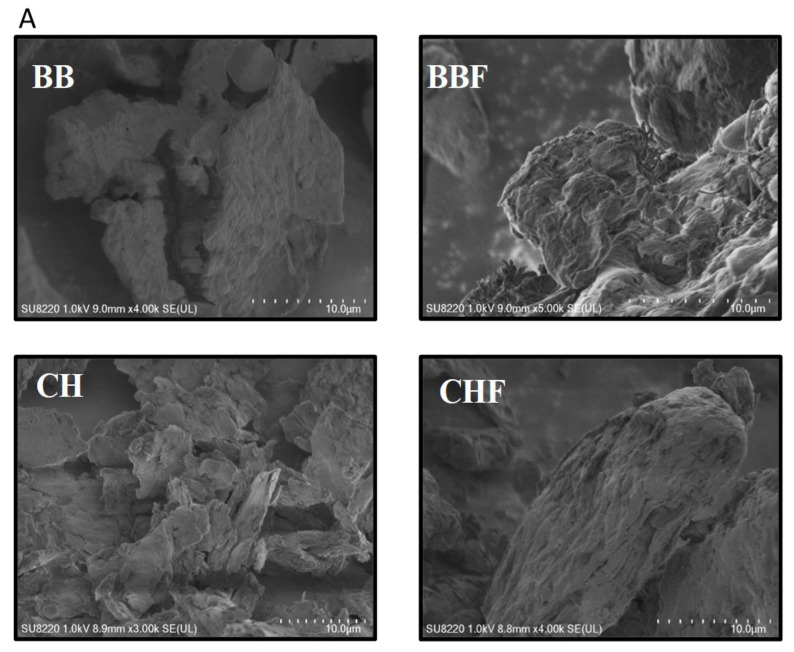

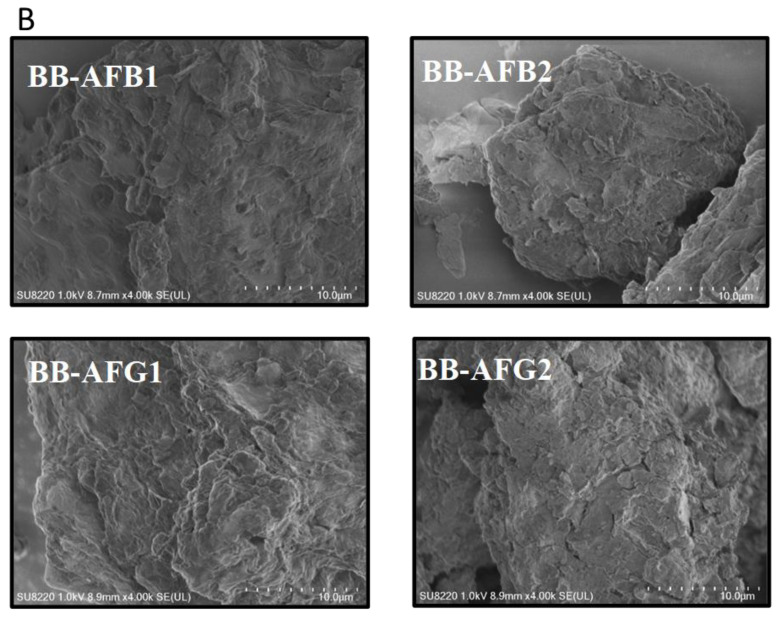
Scanning electron microscope (SEM) images of the fresh adsorbents (**A**) and aflatoxin B1 (AFB1) adsorbed blueberry waste (BB) (**B**).

Scanning electron microscope (SEM) images of the dried and fermented CHs and BBs are displayed in Figure 1 which suggests variable morphology of the four samples. Both types of the biosorbents endured morphological switch after fermentation where untreated BB sample exhibited relatively smooth surface and compact appearance which became highly exfoliated, rougher and looser with large pores and voids after fermentation. The opposite effect of fermentation on the morphologies of BB and CH wastes could be attributed to the amount of adsorbed water and their variable contents hence their decomposition during fermentation might have altered the surface characteristics of both the materials. The SEM imaging of the AFs-adsorbed BB samples presented in Figure 1B showed enhanced surface roughness and etching of the compact structured as compared to those of fresh BB which confirm the successful adsorption of AFs over BB biosorbent.

**Figure 2:**
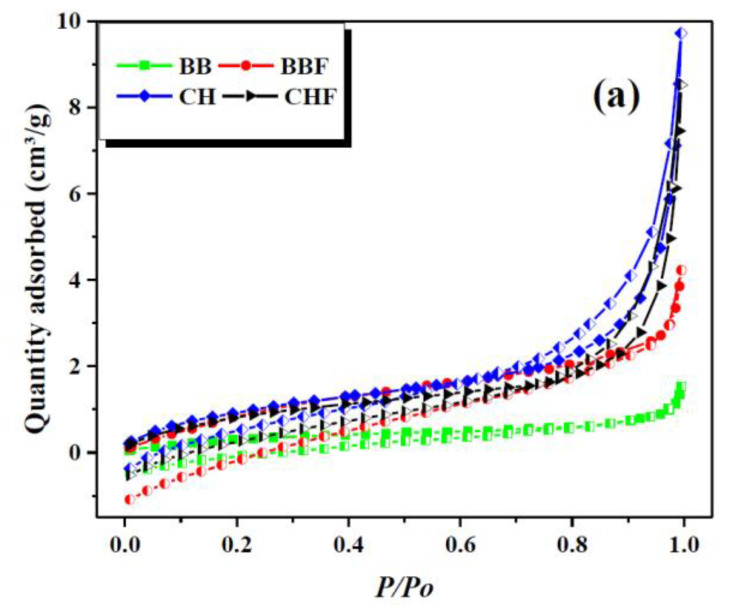

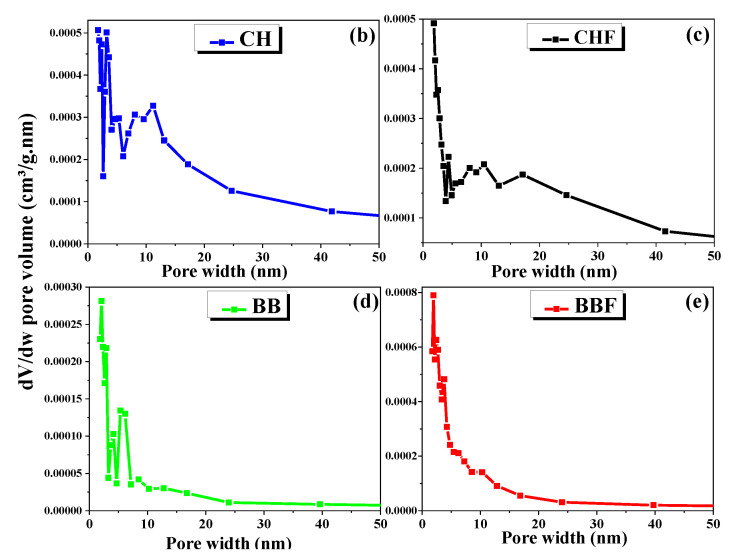
BET surface area analysis (**a**) and pore size distribution of cherry waste (CH) (**b**), cherry fermentation waste (CHF) (**c**), blue berry (BB) (**d**) and blueberry fermentation waste (BBF) (**e**).

Brunauer–Emmett–Teller (BET) surface area and porosity analysis for CH and BB biosorbent were performed to assess the modification resulted from fermentation and the results are shown in Figure 2. 

These results suggest 3.6 times higher surface area and pore volume and smaller pore width and particle size of the fermented BB than the fresh one while no obvious change was observed for CH (Table 1). Moreover, the fermented BB possessed a greater population of micropores as compared to fresh BB sample which might also be responsible for increased BET and specific surface area of pores. These results agreed well with the SEM results where the fermentation enhanced the roughness and porosity of BB biosorbent.

**Figure 3:**
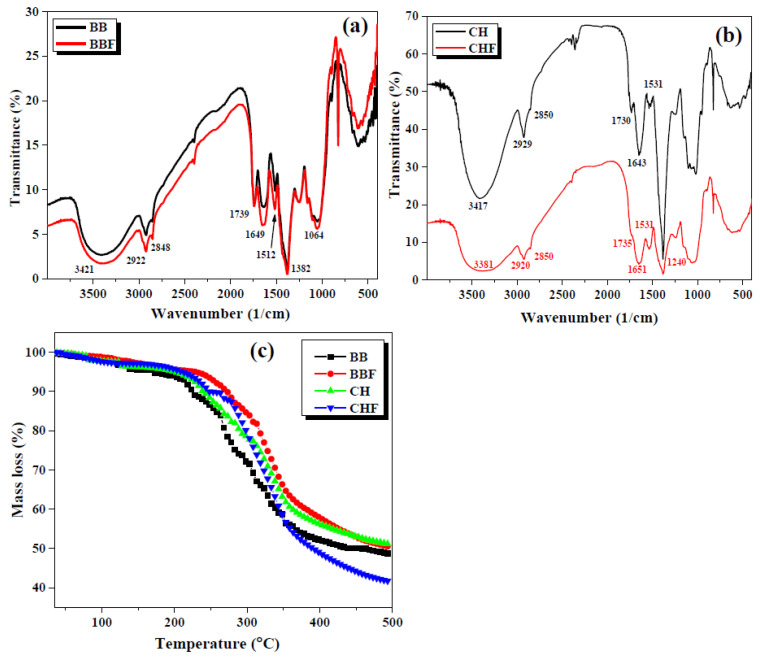
Fourier transform infrared (FTIR) spectroscopy (**a**,**b**) and thermogravimetric analysis (TGA) (**c**) curves of BB, BBF, CH and CHF.

Fourier transform infrared (FTIR) spectroscopy analyses of the biosorbents are displayed in Figure 3a,b. The main peaks in the FTIR spectra of both the fermented and non-fermented BB samples overlapped and appeared at 1064, 1382, 1512, 1649 and 1739 cm^−1^. The absorption band at 1064 and 1382 cm^−1^ could be ascribed to C–OH and C–O–C functional groups respectively [29] whereas bands at 1512, 1649 and 1739 cm^−1^ represent the aromatic C=C, olefinic C=C and C=O functionalities [30]. Peaks at 2848 and 2922 cm^−1^ justified the asymmetric and symmetric stretching vibrations of C–H groups in an organic framework while stretching vibrations of -OH from alcoholic and carboxylic groups were observed around 3421 cm^−1^ as a broad band [31]. Owing to the presumably close chemical composition (mostly cellulosic material) of CH and BB samples, the FTIR spectra of CH and CHF exhibited the major bands very close to the ones observed for BB and BBR.

Thermogravimetric analysis (TGA) curves of the biosorbents are presented in Figure 3c which indicate two stages of weight loss with almost identical thermal degradation trend by all the samples.

During stage one, the mass loss reached up to 10% at 200 °C which would correspond to the evaporation of physically adsorbed water, impurities and volatile organic compounds [32,33]. The second stage showed a rapid decrease in mass loss up to 350 °C which could be attributed to the degradation/pyrolysis of the organic part of the biosorbent [34] and the curves then became constant and moved towards equilibration with a further increase in temperature. These findings suggested the thermally stable nature of the prepared biosorbent in comparison to the operation temperature applied for the AFs adsorption, and hence suggests the feasible nature of the BB biosorbent for practical application.

**Figure 4:**
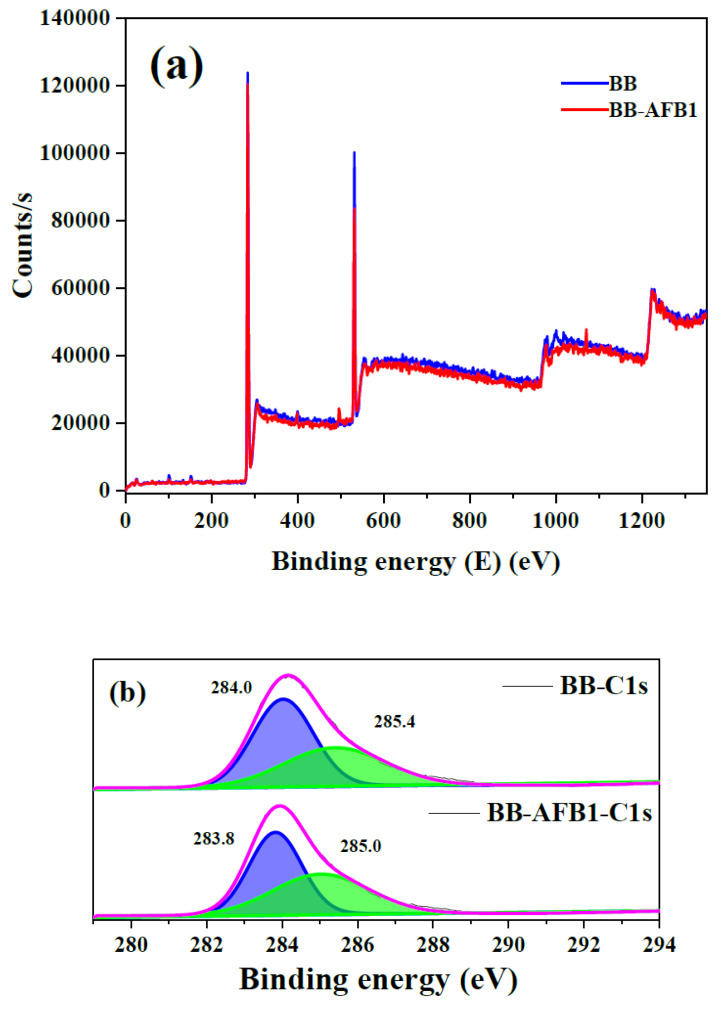

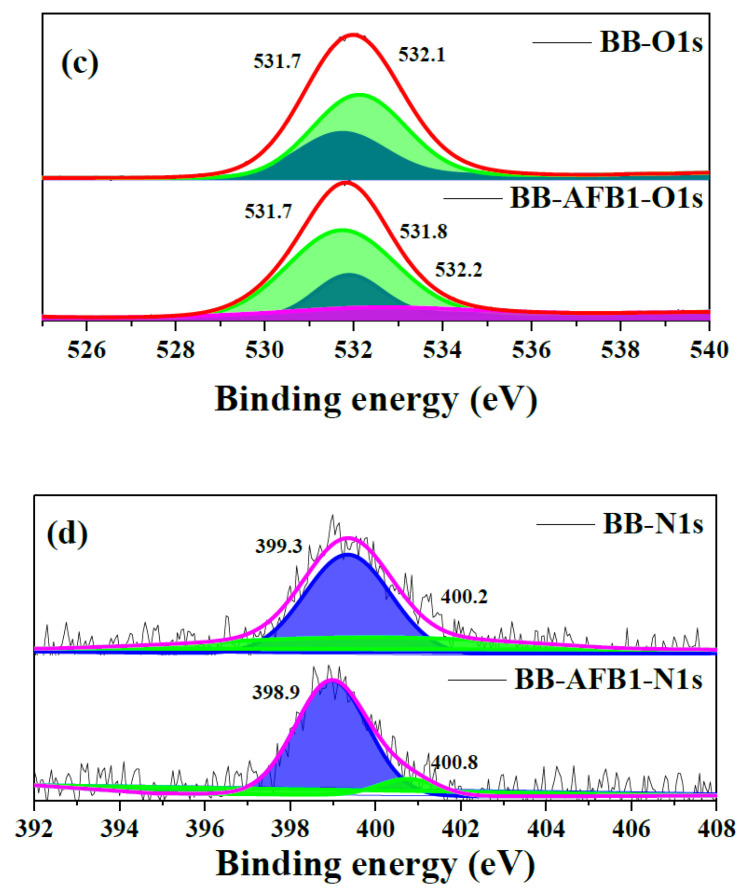
X-ray photon spectroscopy (XPS) full survey (**a**) and high-resolution spectra for C 1s (**b**), N 1s (**c**) and O 1s (**d**) for fresh and AFB1-adsorbed BB.

XPS analysis provides useful information about the surface elemental composition and chemical states [32]. Thus, fresh and AFB1 adsorbed BB samples were analyzed by XPS for surface elemental composition including C, O and N and the results are compiled in Figure 4. Full survey scans clearly indicated high intensity C and O peaks at binding energy (BE) of 284 and 532 eV [35,36] respectively in both BB and BB-AFB1. Very low intensity N peaks were observed at BE of 400 eV and 399 eV for BB and BB-AFB1 respectively [32]. The deconvoluted spectrum of C unraveled two main peaks at 284.0 eV and 285.4 eV for a fresh BB sample, and could correspond to the presence of CC/C=C/CH and CO/CN bonds respectively [18,37] which shifted towards lower BE values in the AFB1-BB samples confirming the successful adsorption of AFs over BB. Similarly, O 1s spectrum was resolved into two peaks at BE of 531.7 eV and 532.1 eV (for both the samples) which mainly depicted the presence of singly and double bonded O to C [32]. However, a new peak emerged in the O 1s spectrum of AFB1-BB sample which could be attributed to the additional chemical states of O atoms present in AFB1 molecules. The N 1s spectrum also exhibited two distinctive peaks at BE of 399.3 eV and 400.2 eV which could correspond to pyridinic N and pyrrolic/pyridonic N atoms [37]. Furthermore, the elemental analysis obtained from XPS showed increased C contents in the AFB1-BB sample as compared to pure BB whereas N and O atoms realized a slight decrease in % atomic contents which could be attributed the chemical structure of AFB1having high C/O ratio and absence of N (Figure S1).

### 2.2. Adsorption Performance of Blueberry

#### 2.2.1. Effect of Solution pH

**Figure 5:**
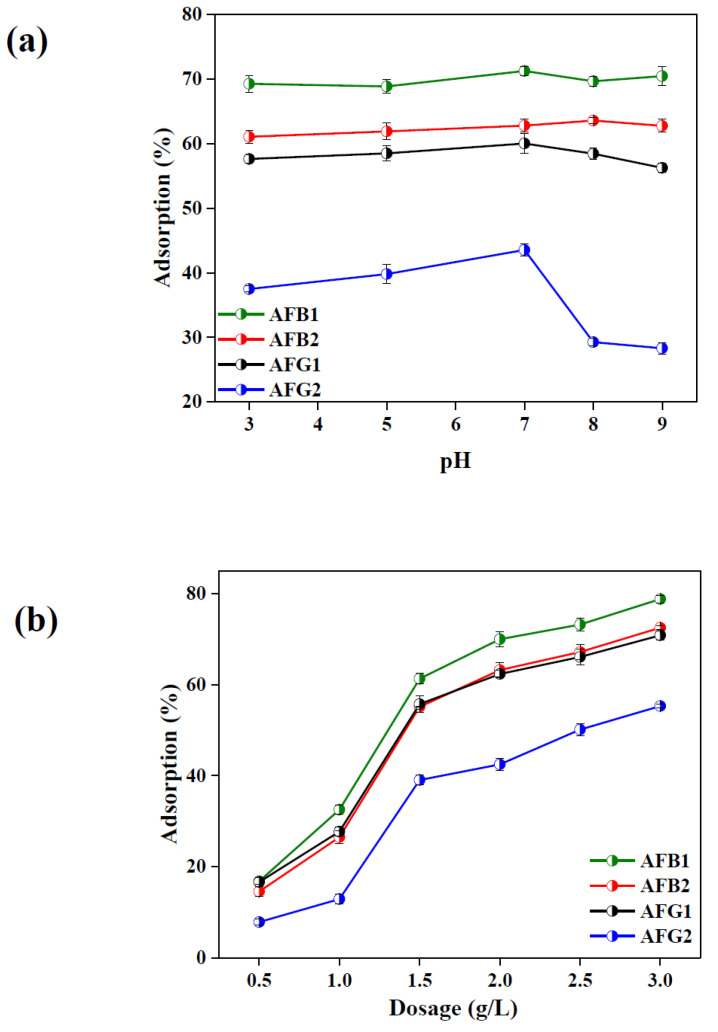
Effect of pH (**a**) and adsorbent dosage (**b**) on aflatoxins (AFs) adsorption onto BB (dosage = 2 mg/mL and C_o_ = 2 ppm for AFB1 and 1 ppm each for AFB2, aflatoxin G1 (AFG1) and AFG2).

pH greatly affects the adsorption process and adsorption capacity in aqueous medium by varying surface charges and adsorbent–adsorbate interactions [38,39]. Therefore, the adsorption capacity of BB for the selected AFs was studied in a pH range of 3.0–9.0. The results compiled in Figure 5a suggest that the adsorption of AFB1 and AFB2 over BB was independent of solution pH whereas adsorption efficiency of AFG1 and AFG2 slightly increased with increasing pH up to 7.0 and then declined in basic medium. This phenomenon could be explained by the net surface charges on BB and the charge on AFs molecules. As AFs carry no charge due the absence of any replaceable protons in their chemical structures (Figure S2) [40] whereas the functional groups on BB became extensively protonated under acidic pH thereby decreasing the adsorption capacity in acidic medium. On the other hand, in basic pH, deprotonation would cause a net negative charge on the BB surface which generates repulsive forces between negatively charged surface of BB and the lone pair carrying O atoms of AFs, which hindered the effective adsorbent–adsorbate interaction and hence led to decreased adsorption performance [41,42]. 

#### 2.2.2. Effect of Adsorbent Dosage

Adsorbent dosage affects adsorption capacity as it can alter the number of active sites on the adsorbent surface. Figure 5b compiles the results of the effect of adsorbent dosage on the adsorption efficiency of BB for the four types of AFs where a continuous increase in % adsorption for AFB1, AFB2, AFG1 and AFG2 was 16–79, 14–72, 16–70 and 7.8–55 respectively was observed by increasing adsorbent dosage from 0.5 g/L to 3.0 g/L. The increase in the adsorption was very sharp initially and onward became milder after achieving equilibrium. This is because increase in dosage causes an increase in the specific surface area and active binding sites necessary for efficient adsorption [35,36]. Therefore, an optimized dosage of 2.0 g/L was selected for further experiments.

#### 2.2.3. Adsorption Kinetics of Aflatoxins over Blueberry

**Figure 6:**
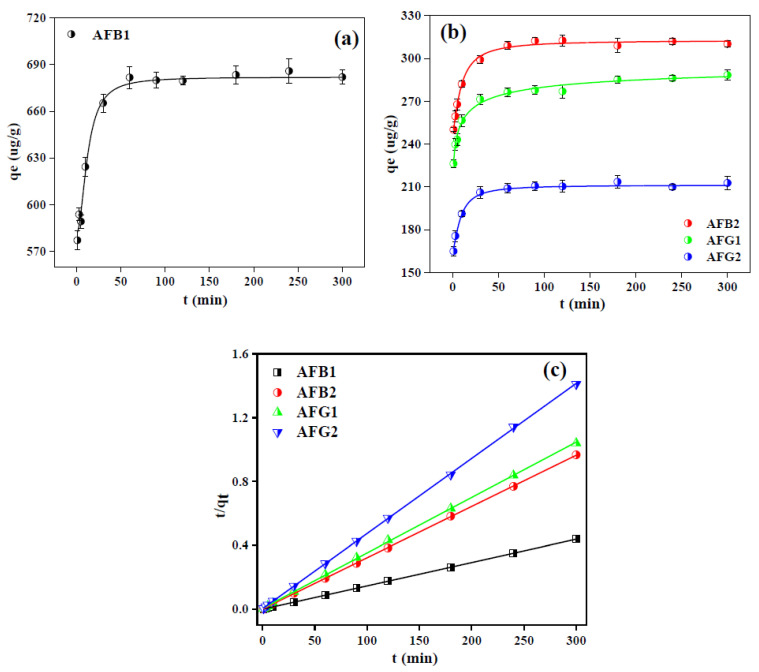
Kinetics study of the adsorption of AFs onto BB (**a**,**b**) and fitting of data with pseudo second order (PSO) kinetics model (**c**) (dosage = 2 mg/mL and C_o_ = 2 ppm for AFB1 and 1 ppm each for AFB2, AFG1 and AFG2).

Kinetics study of the biosorption process is also a factor of great concern as it determines the efficiency and mechanism of adsorption process and, ideally, adsorbent is expected to exhibit fast reaction rate and enhanced adsorption capacity [43]. Figure 6a,b illustrates the adsorption of selected AFs by BB as a function of time and the results exhibited that for all AFs, the equilibrium was achieved within the 60 min of the contact time beyond which no noticeable increase in adsorption capacity was observed. This is because that intake of adsorbate molecules boosted at initial points due to the existence of abundant active binding sites on the adsorbent which reduced with time and finally achieved equilibrium after 60 min [38]. Another important feature is the driving force of mass transfer being provided by the high concentration of AFs molecules at the start of the agitation process [38], which became weaker with the passage of time and hence adsorption performance became slow correspondingly. 

In order to further verify the mechanism behind the adsorption of AFs over BB, pseudo first order (PFO) and pseudo second order (PSO) kinetics models were applied to the kinetic data using Equations (1) and (2) respectively [38,41,44,45,46].

ln (q_e_ − q_t_ ) = lnq_e_ − k_1_t (1)

t/q_t_ = 1/(k_2_ q_e_^2^ ) + t/q_e_ (2)

where qe and qt are the adsorption capacities (mg/g) at equilibrium and at any time t respectively, k1 (min^−1^) and k2 (g.mg^−1^min^−1^) are the PFO and PSO rate constants, respectively. The simulated plots for kinetic models and associated parameters of these models are presented in Figure 6c and Figure S3, and Table 2 respectively. 

Correlation coefficient thus obtained suggested the best suitability of PSO in defining the adsorption mechanism and had R^2^ values above 0.99 for all four types of AFs, while the qe values obtained from PSO were also closer to the experimental qe values than those obtained from the PFO kinetic model. These findings suggested that the chemisorption could be the more prevalent mechanism and rate determining step in the adsorption process [44]. 

#### 2.2.4. Isothermal Behavior of Aflatoxins Adsorption over Blueberry

**Figure 7:**
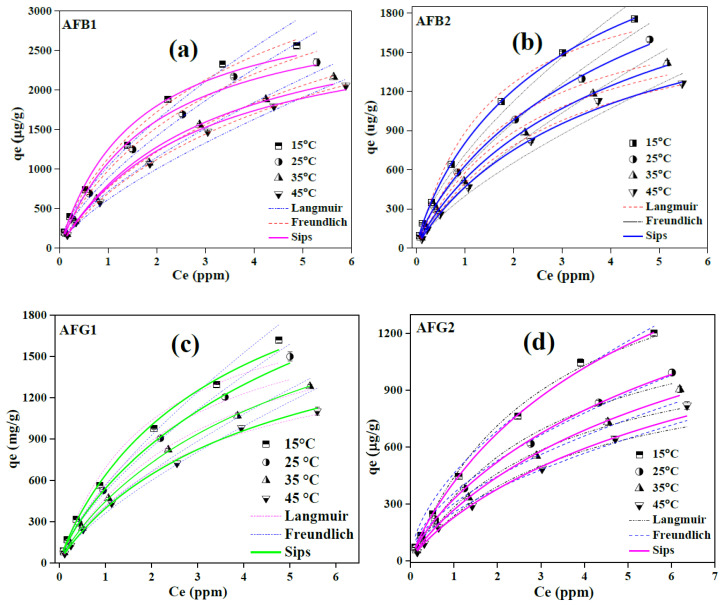
Isothermal study of the adsorption of AFB1 (**a**), AFB2 (**b**), AFG1 (**c**) and AFG2 (**d**) onto BB and the corresponding fitting of data with non-linear regression isotherm models.

Isothermal study is conducted to analyze the interactions between adsorbent and adsorbate and to estimate the maximum adsorption capability of the adsorbent under optimized experimental conditions. Therefore, adsorption isotherms for the four types of AFs were studied at four different temperatures and the experimental data obtained were fitted with Langmuir, Freundlich and Sips using non-linear regression (Figure 7a–d). 

The Freundlich isotherm model describes the reversible and non-ideal adsorption behavior and eliminates the limitation of monolayer adsorption [38,47]. This empirical model relies on a heterogenous and multilayer adsorption process and is described by Equation (3) [48,49].

q_e_ = k_f_ C_e_^(1/n)^ (3)

where, k_f_ (L/mg) and n are Freundlich isotherm constants which signify the biosorption capacity and intensity, respectively.

Langmuir model on the other hand holds best in defining the monolayer coverage on a homogenous adsorbent surface and describes the adsorption process taking place on specific active binding sites of the adsorbent which are identical in terms of adsorption enthalpy and affinity. The non-linear regression of Langmuir isotherm model is given as Equation (4) [50].

q_e_ = (q_m_ k_l_ C_e_)/(1 + k_1_ C_e_) (4)

where k_l_ is the Langmuir isotherm constant and is related to the adsorption energy. Although both Freundlich and Langmuir isotherm models are the most widely used isotherm models to describe the adsorption process, they have limitations to cover all aspects of adsorption process. Hence, the Sips model with three parameters intrinsically covers all the features of both, Freundlich and Langmuir isotherm models and is presented by Equation (5) [47]:

q_e_ = (q_m_ (k_s_ C_e_^(1/ns)^))/(1 + k_s_ C_e_^(1/ns)^) (5)

where k_s_ (L/mg) and n_s_ are the Sips isotherm constant and isotherm exponential coefficient respectively. The corresponding parameters of all the isotherm models for the adsorption of AFs on BB sample (at 15 °C) are compiled in Table 3. The results of models fitting showed that the Sips model was most suitable among the three models in describing the adsorption data with maximum correlation coefficient values of > 0.99 for all types of AFs. As the Sips model is the combined form of Langmuir and Freundlich isotherm model, it could be more suitable in defining one of the isotherm model depending upon the value of the Sips constant k_s_ [47]. If the value of b_s_ is close to 1, the adsorption would be a monolayer, following the Langmuir isotherm model, while its value near to zero employs a Freundlich-type heterogenous adsorption process [47,51]. The obtained b_s_ values for the adsorption of AFs over BB data ranges from 0.77–0.86 (Table 3), suggesting the monolayer adsorption on the homogenous surface of BB samples. Furthermore, the values of n of less than 2.0 obtained for all AFs also suggested the favorable adsorption process on BB. Sips maximum adsorption capacity of BB for AFB1, AFB2, AFG1 and AFG2 were 4.6, 2.9, 2.7 and 2.3 mg/g, respectively, which was higher than many previously reported biosorbents thus suggesting the great promise of BB for the mitigation of AFs form aqueous media. 

#### 2.2.5. Adsorption Thermodynamics

Study of the effect of temperature on adsorption capacity and thermodynamic parameters also provides meaningful evidence of the adsorption mechanism and feasibility of the reaction [43]. The results of the isothermal study conducted at different temperatures concluded that the adsorption capacity was inversely proportional to the temperature, thus revealing the exothermic nature of the adsorption process (Figure 7a–d). The change in Gibb’s free energy (ΔG°), enthalpy (ΔH°) and entropy (ΔS°) were calculated using Equations (6)–(8), respectively [29,43].

ΔG° = −RTlnk_l_ (6)

lnk_l_ = (ΔS°)/R − (ΔH°)/RT (7)

**Figure 8:**
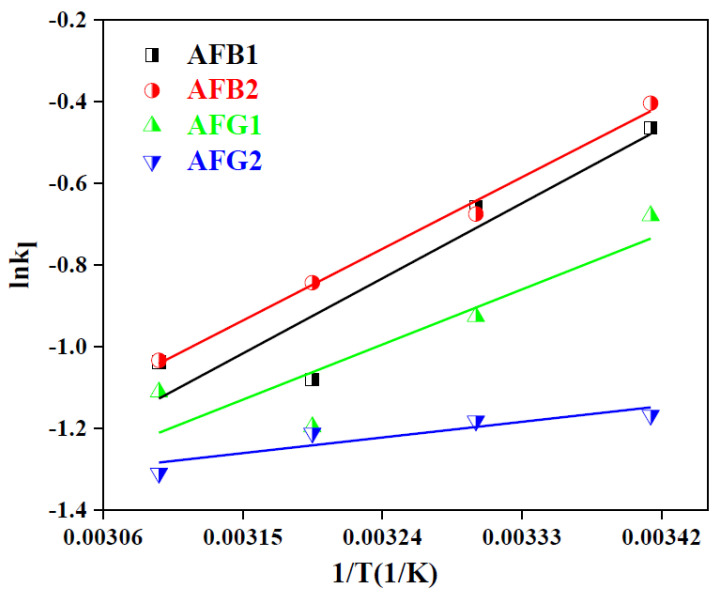
Vant Hoff’s plot for the adsorption of AFs on BB.

ΔG° = ΔH° − TΔS°(8)

where R (8.314 J/K.mol) is the general gas constant and kl is the Langmuir constant at temperature T (K) whereas the values of ΔS° and ΔH° were calculated from the Vant Hoff’s plot (Figure 8) and the resulting thermodynamics parameters are compiled in Table 4. 

The negative values obtained for ΔG° indicated the feasibility of the adsorption process whose magnitude was inversely proportional to the reaction temperature [51]. Likewise, the negative enthalpy values were indicative of the endothermicity of the reaction and negative entropy values indicated that the randomness of the adsorbate molecules at the solid–liquid interface decreased with increasing temperature of the system [29]. Therefore, it was concluded that the adsorption of AFs on the BB sample could be enhanced at low temperatures as reported earlier as well [23]. 

#### 2.2.6. Adsorption in Gastrointestinal Fluids

**Figure 9:**
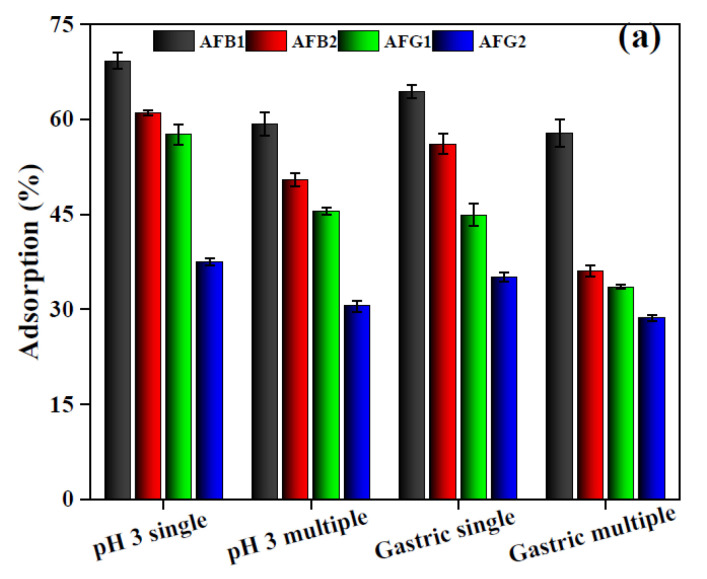

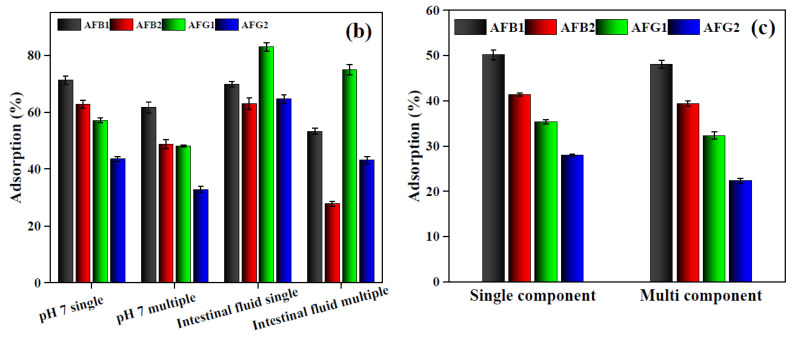
Adsorption of AFs in multi-AFs system and in simulated gastric fluid (**a**) intestinal fluid (**b**) and model wine (**c**) (dosage = 2 mg/mL and C_o_ = 2 ppm for AFB1 and 1 ppm each for AFB2, AFG1 and AFG2).

Batch adsorption experiments were performed in single and multi-AFs system to study their simultaneous removal from gastrointestinal fluid and the results are presented in Figure 9a. where a clear decrease in the adsorption capacity of BB for a multi-AFs system was observed. Likewise, a minor suppression of mycotoxin adsorption capacity was observed for the single component adsorption performed in simulated gastric fluid as compared to a single component buffer system, which declined further for the multi mycotoxin system in simulated gastric fluid. The decrease in the removal efficiency for the multi-mycotoxin system could be justified by the competition of adsorbing species for the same number of active binding sites [52] whereas for gastric fluid, the presence of pepsin might have hindered the adsorption of mycotoxin [53]. This decrease was more pronounced when the adsorption of specific AFs encountered a competitive environment from the coexisting AFs and pepsin simultaneously. 

#### 2.2.7. Simultaneous Adsorption of Aflatoxins

The multiple component adsorption experiments performed at pH 7 also exhibited the same trend of decrease in the adsorption efficiency, however the effect of simulated intestinal fluid had some interesting observations (Figure 9b). Comparison of the single component adsorption in buffer and simulated intestinal solutions did not show any noticeable change in the adsorption efficiency of BB for AFB1 and AFB2 whereas for AFG1 and AFG2, adsorption efficiency increased tremendously. But for multi mycotoxin adsorption performed in simulated gastric fluid, the adsorption of all AFs had a significant decrease which indicate some synergistic or assisting role of bile salts towards AFs adsorption [54].

#### 2.2.8. Adsorption in Model Wine

Some recent studies claimed the predominant presence of AFs in wines and beers [55,56,57,58,59,60] therefore, adsorption experiments of AFs on BB were performed in model wine to estimate the AFs adsorption capacity of BB. The results (Figure 9c) exhibited that the adsorption of all AFs was affected in a wine system as compared to buffer and gastrointestinal solutions in both single and multi-AFs systems. The adsorption carried out in model wine showed around 20% decrease for all AFs as compared to the single component system in buffer solution (pH 3) while this decrease was 22–25% for a multi-component system. This phenomenon could be justified due to the presence of ethanol in the model wine having excellent affinity for less polar AFs and acts as a good solvent for them [61] therefore, affected the generation of effective adsorptive interactions between AFs and BB [61,62]. However, the comparison of the results of single and multicomponent adsorption in model wine showed marginal difference which could probably be due to the existence of distribution coefficient for AFs among liquid and solid phases [48].

### 2.3. Proposed Adsorption Mechanism for Aflatoxins over Blueberry

**Figure 10:**
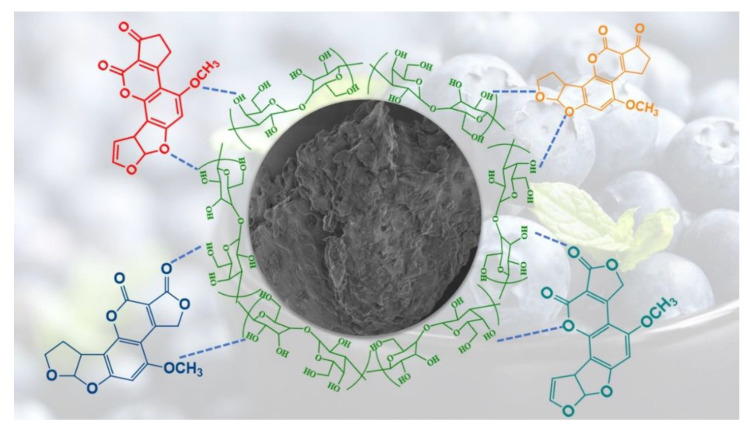
Schematic representation of adsorption mechanism of AFs over BB.

Based on the decoration of agricultural waste with numerous active functional groups (hydroxyl, carbonyl, carboxylic/sulfhydryl carboxylic, phenolic, esters and amino etc.), a possible adsorption mechanism can be proposed for the adsorption of AFs by BB and is shown in Figure 10. 

The presence of plentiful OH groups in the molecular structure of cellulosic/hemi-cellulosic material can establish hydrogen bonds with the O atoms of carbonyl, methoxy and ether groups in AFs molecules, hence providing strong interactive forces for the adsorptive removal of AFs by BB. Moreover, aromatic compounds from the complex BB matrix would also be responsible for π–π stacking [63,64] or the generation of dipole-dipole interactions from O atoms of AFs and organic compounds rich BB. The combination of these multiple interactive forces thereby resulted in the enhanced adsorption performance of BB for the selected AFs both in the simulated and gastrointestinal single as well as multicomponent system, which was far superior than many reported adsorbents (Table 5). 

## 3. Conclusions

In conclusion, this study reports the fabrication of four biosorbents obtained from industrial waste i.e., BB, BBF, CH, CHF which were in turn applied for the sequestration of AFs from multiple matrices, among which CH and BB were found to be superior adsorbents. The synthesized biosorbents were characterized by SEM, FTIR, TGA, BET surface area and XPS techniques while the adsorption of AFs on BB was confirmed by analyzing the post-adsorption BB by SEM imaging and XPS. The adsorption of AFs over BB was marginally affected by solution pH and BB outscored many reported adsorbents in the adsorption capacity for simulated gastrointestinal fluid and model wine. An isothermal study showed the best fitting of adsorption data with the Sips model (Sips maximum adsorption capacity for AFB1, AFB2, AFG1 and AFG2 were 4.6, 2.9, 2.7 and 2.3 mg/g respectively) with a predominant monolayer type adsorption mechanism while kinetics and thermodynamics studies confirmed the rapid AFs uptake and the spontaneity and exothermicity of the adsorption process. The overall coverage of the adsorption performance of BB proved the high competence of this novel, economic, environment-friendly and non-toxic bio-sorbent for the efficient sequestration of AFs and could be envisaged for the remediation of AFs both in vitro and in vivo. However, further studies are required to understand the complex mechanism of the adsorption of AFs on various matrices other than aqueous medium.

## 4. Materials and Methods 

### 4.1. Reagents 

AFs standard solutions were purchased from Sigma-Aldrich ((Shanghai, China). HPLC grade methanol and acetonitrile were supplied by Thermo-Fisher. All other chemicals used for making buffer solutions, gastrointestinal fluid and model wine were of HPLC grade and supplied by Macklin (Shanghai, China). Milli-Q quality water was used for preparing solution and HPLC mobile phase. 

### 4.2. Material Synthesis

Blueberry and cherry fruits were purchased from a local supermarket (Nanning, Guangxi, China), cleaned, washed and divided into two parts i.e., one for juice making and other for fermentation. Fruit pomace was collected by filtration after juice making and washed until the filtrate became transparent. For fermentation, fresh fruits were squeezed and subjected to fermentation according to an already reported procedure [67]. After fermentation, fruit pomace was collected by filtration and washed. Both the pomaces, juice and fermentation pomace, were dried at 60 °C until constant weight and milled to particle size < 75 µm and were kept under an inert environment for the adsorption of AFs.

### 4.3. Characterization of Biosorbents

The chemistry and functional group characterization of the prepared adsorbents were achieved by FTIR using Thermo Fisher Scientific Nicolet 6700 instrument (Thermo Fisher Scientific, Waltham, MA, US) in a range of 400–4000 cm^−1^ following the KBr disc method [68,69,70]. Surface morphology, particle shape and distribution of adsorbent materials were studied by SEM (Hitachi high-technologies corporation, Tokyo, Japan, SU8220, S-3400N) by gold coating the samples prior to analysis. Moreover, the modification of any material brings definite changes in its surface area and porosity characteristics; therefore, to assess the effects of OP extract modification on bentonite, Brunauer–Emmett–Teller (BET) surface area was calculated by N_2_ adsorption-desorption method [71]. Whereas, average particle size, porosity and pore diameter were measured by Barrett–Joyner–Halenda (BJH) method using Mike Instruments (GEMINI VII) (Micromeritics Instrument Corporation, Norcross, GA, USA). Thermogravimetric analysis/derivative thermogravimetry (TGA/DTG) analysis were also performed to have insight in the relative thermal stability of adsorbent materials using NETZSCH TG 209F3 instrument(NETZSCH-Gerätebau GmbH Wittelsbacherstra ße, Selb Germany) in a temperature range of 30–500 °C at a ramp of 10 K/min. XPS analysis was conducted to examine the difference in the elemental composition of adsorbent materials and performed at room temperature using monochromatic Al K alpha radiation (spot size of 650 µm) at an energy step of 1.0 eV (1361 energy steps).

### 4.4. Initial Adsorbent Screening

During initial screening all the four adsorbents i.e., BB juice waste, BB fermentation waste (BBF), CH juice waste and cherry fermentation waste (CHF) were applied for the adsorptive removal of AFs. Adsorption experiments were conducted in 10 mM phosphate buffer saline with an adsorbent dosage of 2 mg/mL and suitable adsorbate concentration (AFB1 (2 µg/mL) and AFB2, AFG1 and AFG2 (1 µg/mL)) at pH 7, 25 °C and 160 rpm. After the adsorption, the supernatant was separated by centrifugation and filtration through 0.22-micron syringe filter. The supernatants were then mixed with properly measured volume of methanol for HPLC quantification. All the adsorption experiments were conducted in triplicates and the results were given as their average value.

### 4.5. High-Performance Liquid Chromatography (HPLC) Quantification of Aflatoxins

A water e2695 HPLC system equipped with Waters fluorescence detector 2475 (λex 365 nm and λem 435 nm) and autosampler was used for AFs quantification. Chromatographic separations of AFs were performed on Agilent reverse phase C18 column (4.6 mm × 250 mm, 5 µm) at 40 °C. A mixture of water:methanol:acetonitrile (60:20:20) was used as mobile phase and pumped at 1 mL/min for 21 min. This method could simultaneously quantify all the under-investigation AFs with a retention time of 9.66, 11.48, 12.78, and 15.35 min for AFG2, AFG1, AFB2, and AFB1 respectively (Figure S4). The adsorption efficiency (%) and the adsorption capacity (qe) were calculated using Equations (**9**) and (**10**) respectively.

Adsorption (%) = (Co − Ce)/Ce × 100(9)

q_e_ = ((Co − Ce)/dose) × V (10)

where Co and Ce represents the initial concentration of toxins to be used for adsorption (before addition of the adsorbent) and the equilibrium AFs concentration left after the completion of adsorption process (µg/mL),V is the volume of the AFs solution (mL) and dose is the mass of the adsorbent (mg).

### 4.6. Batch Adsorption Experiments

After the initial screening of biosorbents, the results (Figure S5), suggested that the BB had superior adsorption capacity for AFs among all four biosorbents tested, therefore, BB was selected to study the effect of different experimental conditions on the adsorptive removal of AFs. To study the effect of pH on the adsorption performance of BB, reactions were performed at pH range of 3–9 with adsorbent dosage of 2 mg/mL and suitable AFs concentrations (AFB1 (2 µg/mL) and AFB2, AFG1 and AFG2 (1 µg/mL)). Adsorption reactions were performed at 25 °C and 160 rpm for 180 min and the residual AFs concentrations were quantified as mentioned earlier. Similarly, adsorbent dosage was optimized in a range of 0.5–3 mg/mL using buffer system of pH 7. Kinetics studies were conducted under similar conditions of buffer system and temperature in the adsorption time range of 1–300 min. 

Isothermal studies were performed at variable temperature i.e., 15–45 °C under optimized reaction conditions i.e., pH 7 and dosage of 2.0 mg/mL with AFs concentration in a range of 1.0–10 mg/mL. The adsorption rate (%) and the adsorption capacity (qe) were calculated using Equations (**9**) and (**10**) respectively. 

### 4.7. Single and Multi-Toxin Adsorption in Simulated Gastrointestinal Fluid

To understand the adsorptive behavior of BB in biological fluids, simulated gastric (pH 2.5) and intestinal fluids (pH 7.0) were prepared according to a previously published method [72], and adsorption was carried out at dosage of 2.0 mg/mL and AFs concentration of AFB1 (2.0 µg/mL) and AFB2, AFG1 and AFG2 (1 µg/ml) at 160 rpm and 37 °C temperature. Adsorption experiments in simulated gastrointestinal fluids were performed in single- and multi-toxin component systems in comparison with buffer system of the same pH. 

### 4.8. Single and Multi-Toxin Adsorption in Model Wine

Model wine was prepared as described elsewhere [73], and adsorption experiments (single and multiple AFs) were carried out as described earlier for gastrointestinal fluid except that temperature was maintained at 25 °C and residual toxin quantification was performed as mentioned earlier. 

## Figures and Tables

**Table 1:** BET surface area and pore size distribution of biomaterials.

Adsorbent	BET Surface Area (m²/g)	Langmuir Surface Area (m²/g)	BJH * Surface Area of Pores (m²/g)	BJH Volume of Pores (cm³/g)	BJH Average Pore Width (nm)	Maximum Pore Volume (cm³/g)	Median Pore Width (nm)	Average Particle Size (nm)
BB	1.41	5.35	1.124	0.002	7.97	0.0004	1.067	4270
BBF	4.59	23.32	3.36	0.006	7.32	0.0011	1.117	1305
CH	3.913	43.41	3.989	0.015	14.66	0.0013	0.988	1533
CHF	3.607	30.5	3.802	0.013	16.555	0.0011	1.005	1663

Note. * represents Barrett-Joyner-Halenda (BJH).

**Table 2:** Kinetic constants of pseudo first-order and pseudo second-order models for the adsorption of aflatoxins on BB.

Type of AF	Pseudo Second Order			Pseudo First Order		
	q_e_ (µg/g)	k_2_ (min^−1^)	*R* ^2^	q_e_ (µg/g)	k_1_ (g/(mg.min))	*R* ^2^
AFB1	1.14E+03	0.00088	0.999	44.53253	0.01217	0.557
AFB2	5.46E+02	0.00183	0.999	21.4246	0.01205	0.713
AFG1	1.66E+02	0.00602	0.999	40.42264	0.01312	0.912
AFG2	2.52E+02	0.00397	0.999	21.25049	0.01095	0.681

**Table 3:** Parameters for Langmuir, Freundlich and Sips isotherms for the adsorption of aflatoxins on BB at 15 °C.

Type of AF	Langmuir Isotherm			Freundlich Isotherm			Sips			
	q_m_ (µg/g)	k_l_ (L/mg)	*R* ^2^	k_f_ (L/mg)	n	*R* ^2^	k_s_	b_s_ (L/mg)	q_s_ (µg/g)	*R* ^2^
AFB1	3229	0.629	0.985	887	1.48	0.989	0.194	0.77	4603	0.995
AFB2	2215	0.668	0.989	718	1.54	0.989	0.229	0.78	2989	0.999
AFG1	2053	0.507	0.989	561	1.38	0.983	0.226	0.86	2716	0.995
AFG2	1860	0.311	0.995	461	1.74	0.988	0.144	0.84	2375	0.999

**Table 4:** Thermodynamics parameters for the adsorption of AFs on BB.

ΔG° (KJ/mol) at (°C)	AFB1	AFB2	AFG1	AFG2
15	−1.531	−1.626	−1.235	−0.758
25	−1.305	−1.282	−0.998	−0.773
35	−0.883	−1.119	−0.786	−0.775
45	−0.951	−0.956	−0.884	−0.725
ΔH° (kJ/mol)	−16.98	−16.21	−12.47	−3.542
ΔS° (kJ/molK)	−0.062	−0.059	−0.049	−0.022

**Table 5:** Comparison of the adsorption capacity of BB with previously reported biosorbents.

Adsorbent Type	Aflatoxin	q_m_ (exp)	Reaction Conditions (Dose and Time)	References
Grape pomace	AFB1	4.7 mg/g	1 g/L, 90 min	[23]
Formosa Firethorn biomass	AFB1	0.016 mg/g	250 g/L, 240 min	[24]
Banana peel	AFB1	0.0084 mg/g	60 g/L, 15 min	[65]
	AFB2	0.0095 mg/g		
	AFG1	0.0004 mg/g		
	AFG2	0.0011 mg/g		
Sangiovese grape pomace	AFB1	2.93 mg/g	2 g/L, 90 min	[66]
Malvasia grape pomace	AFB1	1.43 mg/g	10 g/L, 90 min	
Almond hull	AFB1	2.28 mg/g	15 g/L, 90 min	
Artichoke	AFB1	1.79 mg/g	15 g/L, 90 min	
Blueberry fruit waste	**AFB1**	**4.60 mg/g**	**2 g/L,** **90 min**	**This study**
	**AFB2**	**2.98 mg/g**		**This study**
	**AFG1**	**2.71 mg/g**		**This study**
	**AFG2**	**2.37 mg/g**		**This study**

## Supplementary Materials

The following are available online at https://www.mdpi.com/2072-6651/12/7/466/s1. **Figure S1.** Elemental analysis obtained from XPS analysis; **Figure S2.** Chemical structures of the four types of AFs; **Figure S3.** Pseudo first order kinetics model for the adsorption of AFs onto BB; **Figure S4.** High-performance liquid chromatography (HPLC) chromatogram of multi-AFs system; **Figure S5.** Initial screening of different adsorbents for the adsorption of AFs.
