# Supplementary material for: Assessing the Aflatoxins Mitigation Efficacy of Blueberry Pomace Biosorbent in Buffer, Gastrointestinal Fluids and Model Wine
Source: Toxins (Basel). 2020 Jul 21;12(7):466. doi: 10.3390/toxins12070466 (PMC7405022; doi:10.3390/toxins12070466)
Supplement: Supplementary file 1 [file toxins-12-00466-s001.pdf]

Supplementary Materials: Assessing the Aflatoxins Mitigation Efficacy of Blueberry Pomace Biosorbent in Buffer, Gastrointestinal Fluids and Model Wine

Usman Rasheed, Qurat Ul Ain, Muhammad Yaseen, Sayantan Santra, Xiaohua Yao and Bin Liu

	Atomic % (XPS analysis)		
	**C1s**	**N1s**	**O1s**
**BB**	76.49	1.64	19.29
**BB-AFB1**	78.46	1.31	18.05

Figure S1 Elemental analysis obtained from XPS analysis.

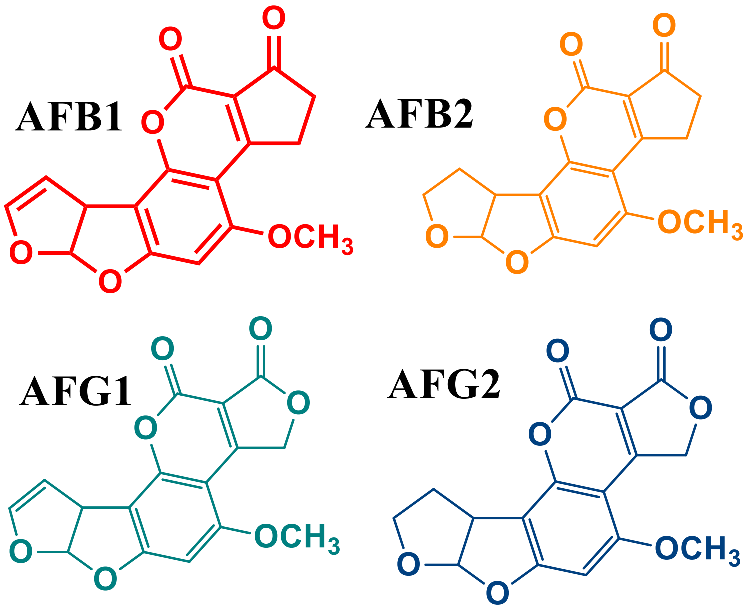

Figure S2. Chemical structures of the four types of AFs.

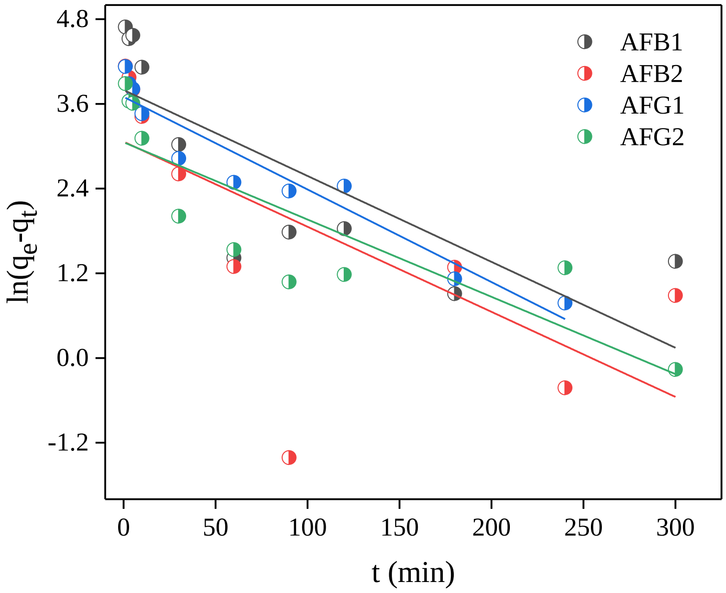

Figure S3. Pseudo first order kinetics model for the adsorption of AFs onto BB (dosage = 2 mg/mL and C_o = 2 ppm for AFB1 and 1 ppm each for AFB2, AFG1 and AFG2).

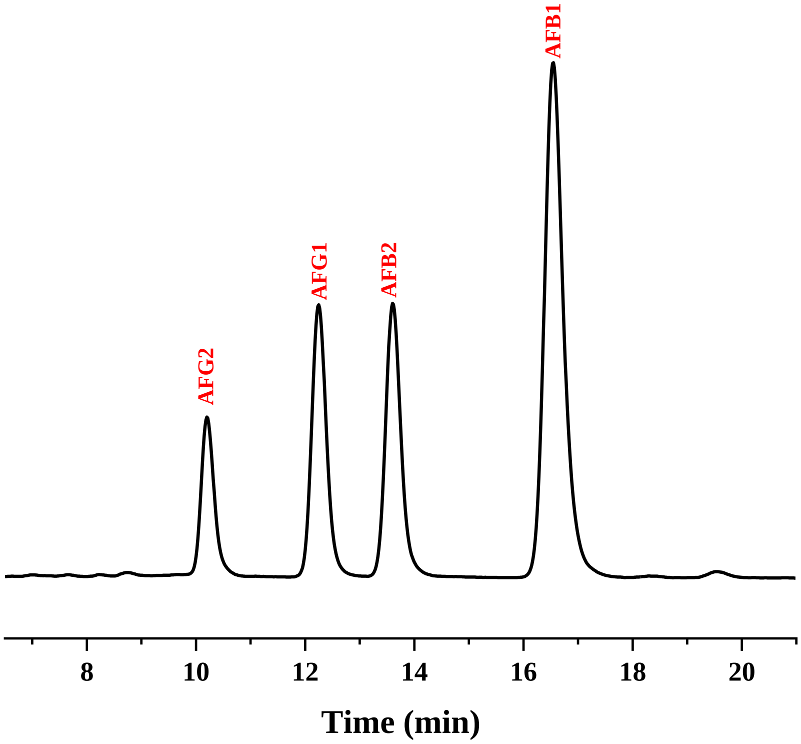

Figure S4 . HPLC chromatogram of multi-AFs system.

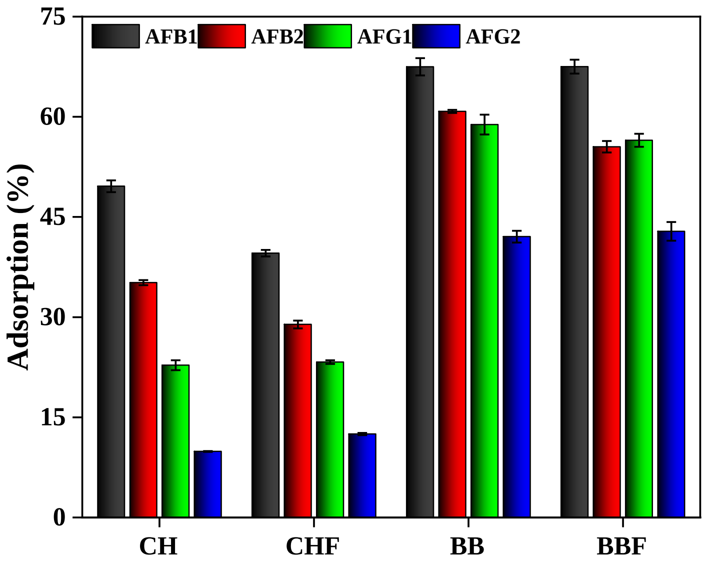

Figure S5. Initial screening of different adsorbents for the adsorption of AFs (dosage = 2 mg/mL and C_o = 2 ppm for AFB1 and 1 ppm each for AFB2, AFG1 and AFG2).
